# Genotype A2/adw2 Strain of Hepatitis B Virus in Turkey

**Published:** 2010-12-01

**Authors:** Murat Sayan, Sila Cetin Akhan, Mitha Bozdayi

**Affiliations:** 1 Clinical Laboratory, PCR Unit, University of Kocaeli, Faculty of Medicine, Kocaeli, Turkey; 2 Department of Clinical Bacteriology and Infectious Diseases, University of Kocaeli, Faculty of Medicine, Kocaeli, Turkey; 3 Institute of Hepatology, University of Ankara, Ankara, Turkey; 4 Gastroenterology Department, Faculty of Medicine, University of Ankara, Ankara, Turkey

**Keywords:** Hepatitis B virus, Genotype

## Abstract

**Background and Aims:**

Previous studies have demonstrated the dominance of genotype D subtype ayw in patients with hepatitis B virus infection in Turkey. The aim of the present study is to report, for the first time, genotype A2 subtype adw2 of hepatitis B virus in a patient who is an inactive hepatitis B carrier in Turkey.

**Materials and Methods:**

Hepatitis B virus DNA isolated from the serum sample was amplified by polymerase chain reaction. The polymerase gene segment of the hepatitis B virus was directly sequenced. A distance matrix/UPGMA comparison was used for phylogenetic analysis, and the genotype of the virus was identified accordingly. The subgenotype and subtype of hepatitis B virus were also detected.

**Results:**

The genotyping of the patient revealed that the isolated hepatitis B virus was genotype A2/adw2.

**Discussion:**

The subtype is inconsistent with the previous data from Turkey; specifically, the identification of the A2/adw2 subtype of the hepatitis B virus in an inactive carrier is the first such case in Turkey. This finding suggests that the transmission of another genotype besides genotype D subtype ayw of the hepatitis B virus is possible in Turkey.

## Introduction

For a DNA virus, the HBV genome shows an exceptional degree of molecular variation (incorporation of some 1010 mistaken nucleotides into the HBV virion per day [1]. Over time, HBV's spontaneous tendency for mutations has led to the emergence of at least eight HBV genotypes (designated A to H), defined by a divergence of 8% or more with reference to the complete nucleotide sequence [2]. These different genotypes show a distinct geographic distribution: genotype A is found in northern Europe, the United States, and Central Africa; B and C predominate in Asia; D is associated with southern Europe, the Middle East, and India; E is uniquely African; and F is found in Central and South America as well as in Polynesia [3][4]. Genotype G has recently been localized in the United States and France [5], whereas a putative eighth genotype H has also been discovered in Central America [6]. Turkey has been found to be an intermediate-endemic region (2% - 7%), with about 6,500 individuals who are newly infected by HBV per year [7][8]. Previous studies have verified the dominance of genotype D subtype ayw in patients with HBV infection in Turkey [7][9][10][11][12]. The aim of the present study is to report, for the first time, that the genotype A2/adw2 of HBV can exist in an inactive hepatitis B carrier patient in Turkey.

## Case Presentation

The patient's condition was detected in a routine screening for amino-acid substitutions for nucleos(t)ide analogues in treatment-naive HBV patients with chronic infection. A 20-year-old male Turkish patient with a significant medical history of nephrotic syndrome and a history of blood transfusion during his childhood was admitted to the hospital. The patient had also been treated for membranous glomerulonephritis in childhood. Following a blood transfusion, the patient had developed hepatitis, nausea, lack of appetite, high Transaminase levels (ALT = 1216 U/L, AST = 903 U/L), and high Bilirubin levels (BU = 9,5 mg/dl, BC = 4,9 mg/dl). Hepatitis B surface antigen (HBsAg), anti-HBcAg total antibody (anti-HBcIgG), and anti-HBeAg antibody (anti-HBe) were later found to be positive. The patient was seronegative for hepatitis A and C viruses.

Because previous serum samples were not available for analysis, the data could not be included in this study (and also the lack of data allowed the patient's condition to change, developing spontaneous HBeAg seroconversion). This case was characterized by HBsAg (+), anti-HBcIgG (+), HBeAg (-), anti-HBe (+) very low HBV DNA load (102 IU/ml), anti-HAV IgG (+), and anti-HAV IgM (-); seronegativity for HDV, HCV, and HIV; normal transaminase levels (ALT = 14 U/L, AST = 19 U/L); and autoantibody negativity (antinuclear, antimitochondrial, antidoublestranded DNA, and antismooth muscle antibodies). The patient had undergone clinical evaluation two times in the last year and had been evaluated as an inactive hepatitis B carrier according to the EASL clinical practice guidelines [3]. Serological markers for HBV were tested using a commercially available microparticle enzyme immunoassay kit (AxSym, Abbott Laboratories, IL, USA). On the other hand, his family members tested negative seronegative for HBV, and the patient reported that he did not travel abroad, use intravenous drugs, or have high risk sexual contact to date.

## Materials And Methods

HBV DNA was isolated from the serum sample on the biorobot workstation using magnetic-particle technology (NucliSENS, easyMAG, bioMérieux, Boxtel, Holland). HBV DNA was detected and quantified by a commercial PCR assay (Iontek Biyotechnology Inc, Istanbul, Turkey) on the real-time platform (iCycler iQ5, Bio Rad Laboratories Inc., California, USA). Briefly, a pair of primers was designed (forward = 5'-TCGTGGTGGACTTCTCTCAATT-3' and reverse = 5'-GTTGACAGACTTTCCAATCAAT-3') for amplification of the HBV polymerase region. The PCR conditions for the polymerase gene segment were as follows: 95°C for 15 min, 45 cycles consisting of 95°C for 45 sec., 56°C for 45 sec., and 72°C for 45 sec. The final concentration of the primer pairs was 0.3 μM. The size of the amplicon of the HBV was around 742-bp. The PCR product was purified using the High Pure PCR Product Purification Kit® (Roche Diagnostics GmbH, Mannheim, Germany) and directly sequenced with the ABI PRISM 310 Genetic Analyzer® equipment using the DYEnamic ET Terminator Cycle Sequencing Kit® (Amersham Pharmacia Biotech Inc, Piscataway, NJ, USA). For cycle sequencing, the following thermal protocol was used: 35 cycles consisting of 95°C for 20 sec., 50°C for 25 sec., and finally 60°C for 2 min. The reverse primer was used as the sequencing primer at a final concentration of 0.5 uM. The electropherogram was assembled using Vector NTI® v5.1 (InforMaxTM InvitrogenTM life science software, Frederick, MD, USA).

**Figure 1:**
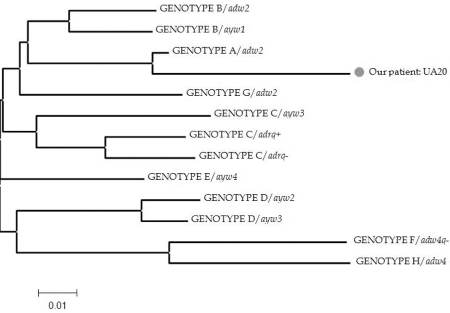
Phylogenetic tree obtained by distance matrix/UPGMA comparison (with Kimura-2 correction) after bootstrapping 1000 replicates of sequence segment at amino acid positions between 80-250 from the reverse transcriptase domain of the polymerase region of hepatitis B virus (UA20 is our patient, the others are from the gene bank)

The HBV genotype was determined by a phylogenetic analysis. The nucleotide sequence was compared to those from the international DNA data bank (DDBJ/EMBL/GenBank). The phylogenetic comparison was performed by a distance matrix/UPGMA analysis using the Kimura 2-parameter via MEGA2 software package program (Figure 1) [14]. However, the HBV genotype was also determined with the genotyping tools of the National Center for Biotechnology Information (NCBI, U.S National Library of Medicine, Bethesda, MD, USA, http://www.ncbi.nih.gov/projects/genotyping), the International Repository for Hepatitis B Virus Strain Data (HepSEQ, http://www.hpa-bioinfodatabases.org.uk), and the Oxford HBV subtype tool (http://www.bioafrica.net/rega-genotype/html/subtypinghbv.html). The subgenotype of the virus was determined as A2 by the Genafor/Arevir geno2pheno drug resistance tool (Center of Advanced European Studies and Research, Bonn, Germany, http://coreceptor.bioinf.mpi-inf.mpg.de). The genotyping and subgenotyping tools work by using BLAST to compare a query sequence to a set of reference sequences for known genotypes.

## Results

The HBV strain was genotyped as A/adw2 (GenBank accession number GU726170) using phylogenetic analysis and genotyping and subgenotyping tools as described above. The similarity of the strain to the subgenotype profile was 98.35% and was therefore subgenotyped as A2.

## Discussion

This case report demonstrates the first identification of the genotype A2/adw2 of HBV in an inactive hepatitis B carrier patient in Turkey. This infection may have been caused by the patient's childhood blood transfusion; it is possible that this may not have been clearly detected via serological methods during the donation of the transfused blood. Several studies have shown that genotype D of HBV is present in almost the entire Turkish patient population infected with HBV [9][10][11][12][15]. According to a restriction fragment length polymorphism study, genotype D with a D2 pattern is the dominant HBV genotype in all age groups in Turkey [16]. It was recently reported that genotype D is still predominant among Turkish patients with chronic HBV infection [17][18]. The analysis of S-gene amino-acid sequences revealed that the surface-gene subtypes of Turkish patients with chronic HBV infection were ayw2 and ayw3 [7]. In another study with a large cohort of Turkish patients with chronic HBV infection, HBsAg subtyping revealed that 99% of the patients were subtype ayw2 [9].

Genotyping of HBV is commonly detected by direct sequencing and phylogenetic tree analysis [7][9].

However, phylogenetic analysis is time consuming for a large number of clinical samples. In our case study, the results were similar for the phylogenetic tree and genotyping tools. Genotyping of HBV on the software programs, such as NCBI, geno2pheno, or HepSEQ genotyping tools is a convenient approach.

Genotyping of HBV has become of great interest as reports indicate that there are differences in the clinical outcome of infection by genotype and differences in response to treatment. For instance, HBV genotypes A and B have been shown to be associated with a better response to interferon alpha than genotypes C and D. However, HBV genotype does not influence the response to any nucleos(t)ide analogues [13].

In conclusion, our case is the first case in which the genotype A2/adw2 of HBV was detected, and this is not consistent with previous data from Turkey. This finding suggests that transmission of another genotype, except genotype D subtype ayw of HBV, is possible in Turkey.
